# Delayed post-diuretic ^18^F-FDG PET/CT for preoperative evaluation of renal pelvic cancer

**DOI:** 10.7150/jca.44512

**Published:** 2020-04-06

**Authors:** Yiping Shi, Ruohua Chen, Yining Wang, Gan Huang, Qian Xia, Jianjun Liu

**Affiliations:** Department of Nuclear Medicine, Ren Ji Hospital, School of Medicine, Shanghai Jiao Tong University, Shanghai, China

**Keywords:** PET/CT, renal pelvic cancer, SUVmax

## Abstract

**Background**: Application of ^18^F^-^fluorodeoxyglucose positron emission tomography (^18^F-FDG PET) in urological oncology was relatively slowly due to the urinary elimination of ^18^F-FDG. We investigated whether delayed post-diuretic ^18^F-FDG PET/CT could be used for diagnosing renal pelvic cancer.

**Methods**: 51 patients were included who underwent delayed post-diuretic ^18^F-FDG PET/CT for detecting renal pelvic space-occupying lesions. The comparations of delayed PET/CT parameters and clinical characteristics between renal pelvic cancer and benign polyp were investigated.

**Results**: Among the 51 patients, 47 were found to have renal pelvic urothelial carcinoma, and 4 had benign polyp. ROC analysis identified the lesion maximum standardized uptake value (SUVmax) of 6.2 as the optimal cut-off value to distinguish from renal pelvic urothelial carcinoma to benign polyp. With the SUVmax cut-off of 6.2, the sensitivity, and specificity for predicting of renal pelvic urothelial carcinoma were 91.5% (43/47), and 100% (4/4). We also found a significant difference in tumor size between the positive (SUVmax > 6.2) and negative (SUVmax ≤ 6.2) PET groups in renal pelvic cancers. In patients with tumor size < 1.1 cm, the probability of being in the negative PET group was 75%. In such patients, a substantial proportion of renal pelvic cancer demonstrated negative SUVmax similar to that in patients with benign polyp.

**Conclusion**: Delayed ^18^F-FDG PET/CT could be used for differentiating renal pelvic cancer from benign polyp. In patients with small tumor size, renal pelvic cancer may present low ^18^F-FDG uptake, mimicking the metabolic phenotypes of patients with benign polyp.

## Introduction

Renal pelvic cancer is relatively uncommon urological malignancies 1, 2. Urothelial carcinoma is the most common histologic type of renal pelvic cancer 3, 4. Magnetic resonance imaging and computed tomography are routinely used for diagnosis of renal pelvic cancer 5, 6. ^18^F^-^fluorodeoxyglucose (FDG) positron emission tomography (PET) has been widely used in many malignant tumors 7, 8. However, application of ^18^F-FDG PET/CT specifically in urological oncology is relatively slowly due to the urinary elimination of ^18^F-FDG 9, 10. It is encouraging to note that ^18^F-FDG PET/CT is used in the evaluation of bladder cancer through delayed post-diuretic imaging and has exhibited high sensitivity and accuracy in several studies 11-14. However, few studies have examined the role of ^18^F-FDG PET/CT in the diagnosis of renal pelvic cancer. So far, whether delayed post-diuretic ^18^F-FDG PET/CT could be used for evaluation of renal pelvic cancer and the accuracy remains unclear.

The purpose of our study was to assess the value of delayed post-diuretic ^18^F-FDG PET/CT in the diagnosis of renal pelvic cancer and to distinguish between renal pelvic cancer and benign polyp. In addition, we also assessed the clinicopathologic features which were correlated with ^18^F-FDG uptake and identify the patients with renal pelvic carcinoma who may demonstrate negative ^18^F-FDG PET/CT results.

## Methods

### Patients

We retrospectively reviewed 51 patients with renal pelvic space-occupying lesions who were examined by delayed post-diuretic ^18^F-FDG PET/CT at the Shanghai Jiaotong University-affiliated Ren Ji Hospital from January 2012 to July 2019. Among the 51 patients, 41 patients were treated with nephroureterectomy; the remaining 10 patients were treated with ureteroscopy and biopsy. The pathological results were acquires using the specimen obtained from nephroureterectomy (n=41), or ureteroscopy (n=5) and biopsy (n=5). RenJi Hospital institutional review board approved this study. The informed consent was waived because it was a retrospective study.

### Delayed post-diuretic ^18^F-FDG PET/CT imaging

After fasting for at least 6 h, the patients received an intravenous 3.7 MBq/kg injection of ^18^F-FDG. ^18^F-FDG PET/CT scanning was carried out using a whole-body scanner (Biograph mCT; Siemens) (early PET/CT imaging). After early PET/CT imaging, delayed post-diuretic PET/CT imaging was carried out after 120 min of early PET/CT imaging. Patients received 20 mg of furosemide by the oral route and an oral intake of more than 500 mL water. Patients were required to void frequently to reduce the urine physiological uptake of the radiotracer ^18^F-FDG. Delayed PET/CT comprised a range of two bed positions centered at the location of the renal pelvis. Regions of interest were placed over the most intense area of ^18^F-FDG uptake on delayed post-diuretic PET/CT imaging to calculate the SUVmax.

### Statistical analysis

The data are used as mean ± SD. The relationship between the clinicopathological characteristics and renal pelvic carcinoma and benign polyp were analysed by the chi-square test, Fisher's exact test or Mann-Whitney U test, where applicable. Receiver operating characteristic (ROC) curve analysis was performed to determine the cut-off values for differentiating renal pelvic cancer from benign polyp. P<0.05 was considered statistically significant. SPSS, version 13.0 (SPSS Inc.) was used to perform statistical analyses.

## Results

### Patient characteristics

**Figure 1:**
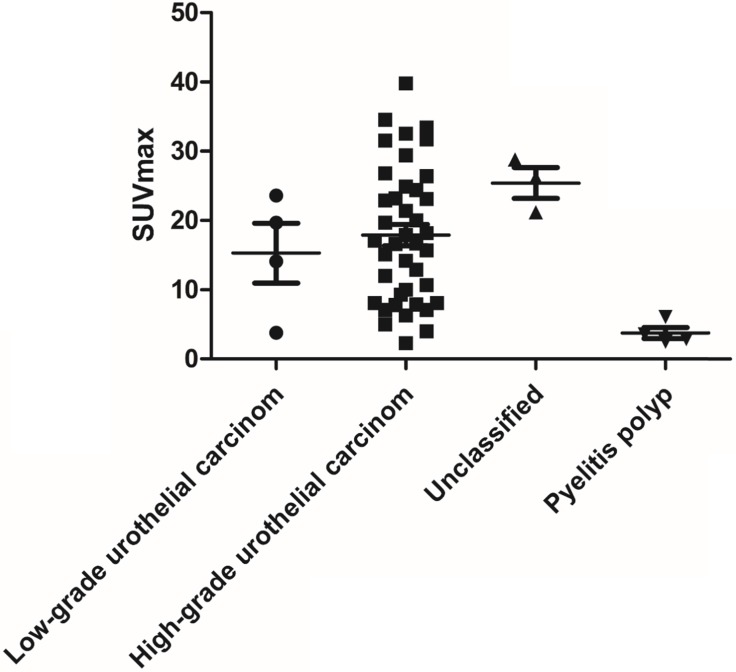
The scatter plot of SUVmax for each urothelial carcinoma grade and benign polyp.

**Figure 2:**
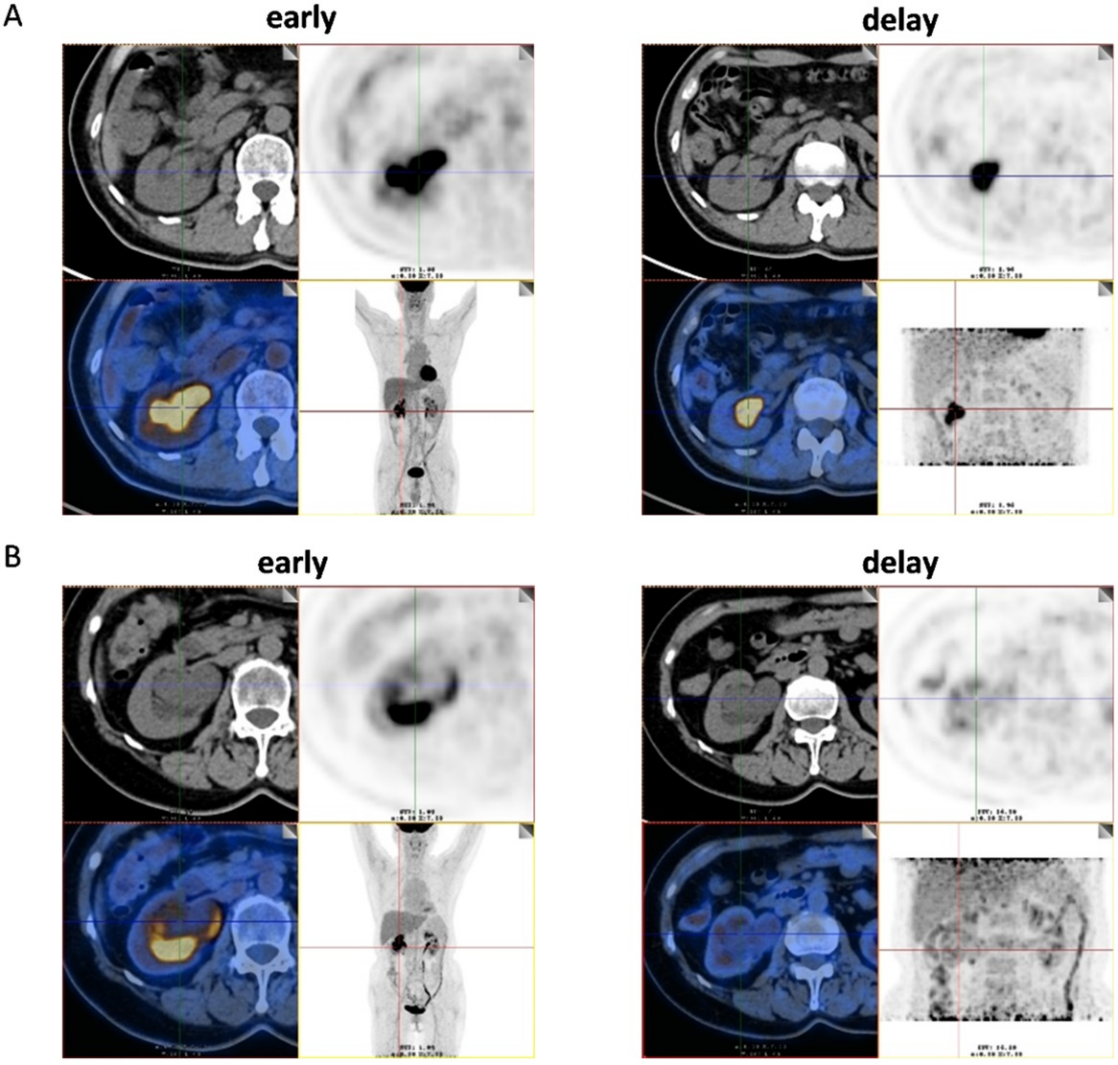
** (A)** Images of a 56-year-old man with renal pelvic cancer with high SUVmax (SUVmax=14.1). On the ^18^F-FDG PET/CT images, axial CT show that the space-occupying lesion was located in right renal pelvis. However, the ^18^F-FDG uptake of lesion cannot be easily detected because of urine interference in the early image (early). On the image of ^18^F-FDG PET/CT in the delayed phase, the ureter is distended and lesion can be easily visualized by axial CT and PET (SUVmax, 14.1) (delay) as the ^18^F-FDG uptake of urine was very low. The patient underwent radical resection of renal pelvic cancer, and low-grade urothelial carcinoma was confirmed by histopathology. **(B)** Images of a 61-year-old woman with benign polyp with low SUVmax (SUVmax=2.9). On the ^18^F-FDG PET/CT images, axial CT show that the space-occupying lesion was located in right renal pelvis. However, the ^18^F-FDG uptake of lesion cannot be easily detected because of urine interference in the early image (early). On the image of ^18^F-FDG PET/CT in the delayed phase, the ureter is distended and lesion can be easily visualized by axial CT and PET (SUVmax, 2.9) (delay) as the ^18^F-FDG uptake of urine was very low. The patient underwent pyeloureterectomy, and benign polyp was confirmed by histopathology.

Among the 51 patients, 47 had malignant tumors: these included 40 cases of high- grade urothelial carcinoma, 4 cases of low-grade urothelial carcinoma and 3 cases of unclassified urothelial carcinoma. The remaining 4 patients had benign lesions, which were confirmed as benign polyp. Patient characteristics were shown in Table 1. The scatter plot of maximum standardized uptake value (SUVmax) for each urothelial carcinoma grade and benign polyp is shown in Figure 1. Mean SUVmax for high-grade urothelial carcinoma, low-grade urothelial carcinoma and benign polyp were 17.9 ± 9.7, 15.3 ± 8.6 and 3.8 ± 1.6, respectively. Both high-grade urothelial carcinoma (17.9 ± 9.7 vs 3.8 ± 1.6, P < 0.001) and low-grade urothelial carcinoma (15.3 ± 8.6 vs 3.8 ± 1.6, P = 0.039) showed significantly higher SUVmax compared with the benign polyp. However, there was no significant difference in SUVmax between high-grade urothelial carcinoma and low-grade urothelial carcinoma (17.9±9.7 vs 15.3 ± 8.6, P = 0.651). Representative images of a patient with renal pelvic carcinoma with high SUVmax (SUVmax=14.1) (Fig. 2A) and a patient with benign polyp with low SUVmax (SUVmax=2.9) (Fig.2B) who underwent early PET/CT imaging and delayed post-diuretic PET/CT scanning were shown in Figure 2.

The mean SUVmax of normal kidney tissues from all patients was calculated as 4.4 ± 1.2, and it was not significantly different urothelial carcinoma grades and benign polyp (Kruskal-Wallis test; P = 0.337); therefore, this value was used as the control SUVmax. The rates of positive ^18^F-FDG accumulation were significantly higher in patients with renal pelvic cancer than in benign polyp (95% [44/47] vs. 25% [1/4], respectively; P = 0.004) when compared with normal kidney tissues.

### Differences ^18^F-FDG PET/CT parameters between renal pelvic carcinoma and benign polyp

Table 2 depicts patients' characteristics and delayed ^18^F-FDG PET/CT imaging grouped on the basis of renal pelvic carcinoma and benign polyp. No significant intergroup differences were found in terms of sex, age, primary site laterality, and lesion size. However, significant intergroup differences were found with respect to SUVmax. To elaborate, renal pelvic carcinoma had higher SUVmax than benign polyp (17.6 ± 11.5 vs. 4.5 ± 2.8; P < 0.001).

### Measurement of SUVmax cut-off value

**Figure 3:**
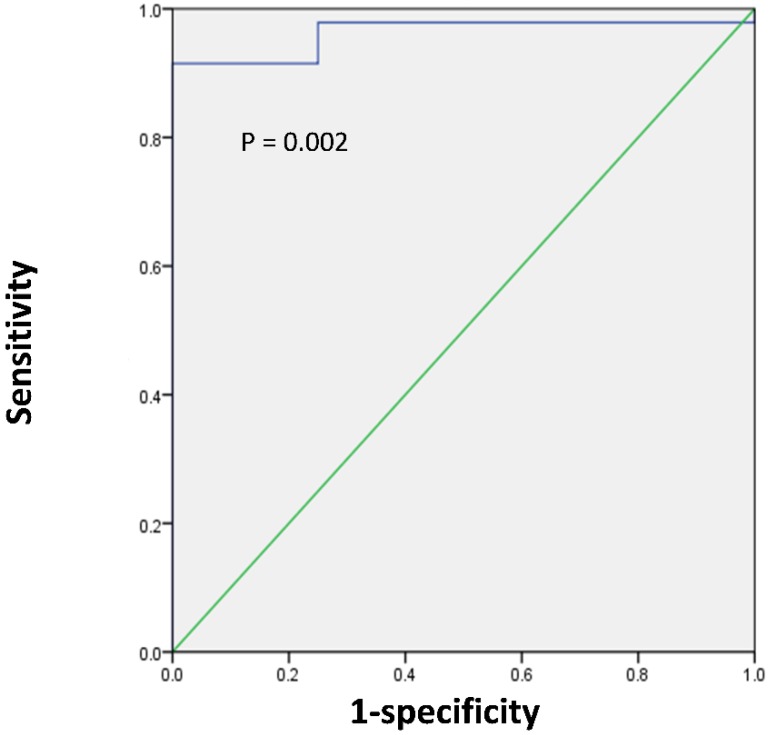
ROC curve analysis for the differentiating renal pelvic cancer from benign polyp according to the SUVmax of lesion in 51 patients with renal pelvic space-occupying lesions. The area under the curve was 0.963 (95%CI 0.909-1.0, P = 0.002), and 6.2 was determined as the best SUVmax for predicting renal pelvic cancer. With an SUVmax cut-off of 6.2, the sensitivity, specificity, positive predictive value (PPV), and negative predictive value (NPV) for the prediction of renal pelvic carcinoma were 91.5% (43/47), 100% (4/4), 100% (43/43), and 50.0% (4/8), respectively.

Next, we sought to determine the optimal SUVmax for differentiation between renal pelvic carcinoma and benign polyp. ROC analysis showed that the highest accuracy (92.2%) was obtained with an SUVmax cutoff of 6.2 and that the area under the curve was 0.963 ± 0.028 (Figure 3). With an SUVmax cut-off of 6.2, the sensitivity, specificity, positive predictive value, and negative predictive value for the prediction of renal pelvic carcinoma were 91.5% (43/47), 100% (4/4), 100% (43/43), and 50.0% (4/8), respectively.

In the multivariate analysis including factors with a P value of 0.15 or less, SUVmax remained the independent prognostic factor for differentiation between renal pelvic carcinoma and benign polyp [Table 3; OR, 2.76; 95% CI, 1.01-7.545; P = 0.048].

### The relationships between clinicopathologic features and ^18^F-FDG PET/CT results in renal pelvic cancers

Of the 47 renal pelvic cancers, 38 patients were treated with radical resection of renal pelvic cancer. We further evaluated the associations between clinicopathologic features and delayed PET/CT results in these 38 patients by univariate analysis (Table 4). According to the SUVmax of tumors, the patients were divided into positive PET group (SUVmax > 6.2) and negative PET group (SUVmax ≤ 6.2). No significant intergroup differences were found inclung sex, age, tumor laterality, TNM stage, and lymph node metastasis. However, the tumor size was significantly larger in the positive PET group than the negative PET group (32.1 ± 17.4 vs. 8.7 ± 2.3; P = 0.027).

**Figure 4:**
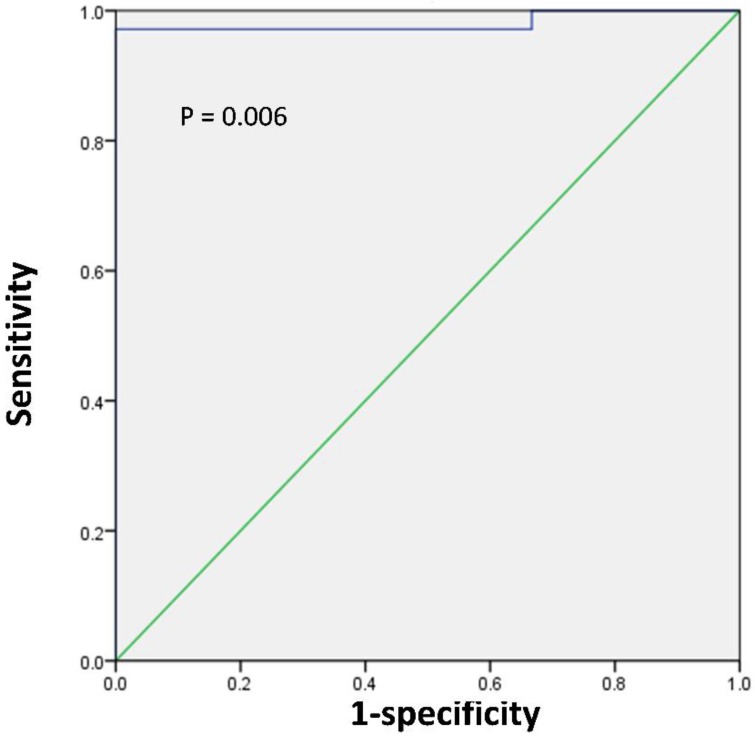
ROC curve analysis for predicting tumor SUVmax in positive or negative PET group according to the tumor size in 38 patients with renal pelvic cancer. The area under the curve was 0.981 (95%CI 0.938-1.0, P = 0.006), and 1.1 cm was determined as the best tumor size for predicting tumor SUVmax in positive or negative PET group. With the tumor size of 1.1 cm, the sensitivity, specificity, positive predictive value, and negative predictive value for predicting tumor SUVmax in positive PET group were 97.1% (34/35), 100% (3/3), 100% (34/34), and 75% (3/4), respectively.

Next, we sought to determine the tumor size threshold for predicting tumor SUVmax in positive or negative PET group. ROC analysis demonstrated that the highest accuracy (97.4%) was obtained with the tumor size cutoff of 1.1 cm and that the area under the curve was 0.981 ± 0.022 (Figure 4). With the tumor size of 1.1 cm, the sensitivity, specificity, positive predictive value, and negative predictive value for predicting tumor SUVmax in positive PET group were 97.1% (34/35), 100% (3/3), 100% (34/34), and 75% (3/4), respectively.

## Discussion

^18^F-FDG PET/CT is used for diagnosis of many malignant tumors 15, 16. Unfortunately, ^18^F-FDG is not an ideal radiotracer for use in urology due to its urinary elimination 12. In our study, the application of delayed post-diuretic ^18^F-FDG PET/CT for diagnosing renal pelvic space-occupying lesions was analyzed. So far, our study was the first to demonstrate that delayed post-diuretic ^18^F-FDG PET/CT could be used for diagnosing renal pelvic cancer and distinguishing between renal pelvic cancer and benign polyp.

Although most of renal pelvic space-occupying lesions are renal pelvic cancer, it should also be distinguished from other renal pelvic space-occupying benign lesions 17. Early differentiation between renal pelvic cancer and benign polyp is crucial for executing an effective treatment plan and predicting prognosis. Although magnetic resonance imaging and computed tomography have been widely used for the diagnosis of renal pelvic cancer, its accuracy for distinguishing between renal pelvic cancer and benign polyp is limited; in addition, ureteroscopy, which requires general anesthesia, is an invasive method that carries the risk of infection 18, 19. Our study found that delayed post-diuretic ^18^F-FDG PET/CT could be used to differentiate renal pelvic cancer from benign polyp. The ROC analysis indicated that SUVmax could potentially be used to predict whether the renal pelvic space-occupying lesions is malignant or benign polyp. Multivariate analysis demonstrated that the SUVmax of the lesion was the significant predictor of the nature of the renal pelvic space-occupying lesions. Our study is the first to conclude that the SUVmax of the renal pelvic space-occupying lesions could be used to differentiate between renal pelvic cancer and benign polyp. With an SUVmax cut-off of 6.2, the sensitivity and specificity for the prediction of renal pelvic cancer were 91.5% and 100%, respectively. Thus, lesions with SUVmax greater than 6.2 indicate a high possibility of renal pelvic cancer.

In this study, we further analyzed the relationships between clinicopathologic features and delayed post-diuretic^ 18^F-FDG uptake in renal pelvic cancers. Our findings suggest that tumor size was significantly correlated with the SUVmax of renal pelvic cancer; whereas other feathers such as age, sex, tumor laterality, tumor grade and TNM stage do not play an important role in determining the SUVmax of tumors. We found that when patients had tumor size < 1.1 cm, their probability of being in the negative PET group was 75%. In such patients, a substantial proportion of renal pelvic cancer demonstrated low ^18^F-FDG uptake similar to that in patients with benign polyp. Therefore, when using SUVmax to distinguish renal pelvic cancer from benign polyp, the lesion size should also be considered, especially in the negative PET results. For patients with lesion size < 1.1 cm, even if the SUVmax of renal pelvic space-occupying lesions was relatively low (SUVmax < 6.2), we cannot arbitrarily assume that it is benign polyp.

Our study was its limitation because it was a retrospective study and the sample size was relatively small. Although delayed post-diuretic ^18^F-FDG PET/ CT may have a good diagnostic performance, in the clinical setting it is not possible to establish a threhold for SUVmax, and delayed post-diuretic ^18^F-FDG PET/CT could not replace ureteroscopy, biopsy, post-operative histopathology for determining the lesion nature of renal pelvic space-occupying lesions.

## Conclusions

Our results were the first to show the application of delayed post-diuretic ^18^F-FDG PET/CT in the renal pelvic space-occupying lesions and demonstrated that delayed ^18^F-FDG PET/CT could be used for differentiating renal pelvic cancer from benign polyp. These results may advance the development of noninvasive methods to predict benign and malignant disease of renal pelvic space-occupying lesions. Further larger and prospective studies that include more clinical samples are needed to confirm the value and efficacy of delayed ^18^F-FDG PET/CT in the renal pelvic space-occupying lesions.

## Figures and Tables

**Table 1:** Patient characteristics (n=51)

Histology and Grade	No. Patients (n)	Age (year)	Male: Female (n)
**Renal pelvic carcinoma**			
High-grade urothelial carcinoma	40	65.1±10.3	21:19
Low-grade urothelial carcinoma	4	70.3±9.5	3:1
Unclassified urothelial carcinoma	3	68.0±8.9	3:0
**Benign lesions**			
Benign polyp	4	48.0±11.5	1:3

**Table 2:** Patient characteristics according to renal pelvic carcinoma and benign polyp (n=51)

Characteristics	Total (n=51)	Renal pelvic carcinoma (n=47)	Benign polyp (n=4)	P
**Age (y)**				
≤60	16	13	3	0.086
>60	35	34	1	
**Sex**				
Male	28	27	1	0.316
Female	23	20	3	
**Lesion laterality**				
Right	26	23	3	0.61
Left	25	24	1	
**Lesion Size**		31.3 ± 17.6	16.5± 10.5	0.117
**SUVmax**		18.2 ± 9.5	3.8 ± 1.6	<0.001

**Table 3:** Multivariate analysis for differentiation between renal pelvic carcinoma and benign polyp

Factors	Odds Ratio	OR (95% CI)	P
**SUVmax**	2.76	1.01-7.545	0.048
**lesion size**	0.894	0.744-1.074	0.231
**age**	162.48	0.217-121899.462	0.132

**Table 4:** Patient characteristics according to positive and negative PET group (n=38)

Characteristics	Total (n=38)	Positive	Negative	P
**Age**				
≤60	10	9	1	0.774
>60	28	26	2	
**Sex**				
Male	19	18	1	0.547
Female	19	17	2	
**Tumor laterality**				
Right	19	18	1	0.547
Left	19	17	2	
**Tumor Size (mm)**		32.1±17.4	8.7±2.3	0.027
**TNM stage**				
0	18	15	3	0.097
2-4	20	20	0	
**Lymph node metastasis**				
0	30	31	3	0.536
1	8	4	0	
